# A customized framework for regional classification of conifers using automated feature extraction

**DOI:** 10.1016/j.mex.2021.101379

**Published:** 2021-05-10

**Authors:** Cali L. Roth, Peter S. Coates, K. Benjamin Gustafson, Michael P. Chenaille, Mark A. Ricca, Erika Sanchez-Chopitea, Michael L. Casazza

**Affiliations:** U.S. Geological Survey, Western Ecological Research Center, Dixon, CA, United States

**Keywords:** Automated feature extraction, Classification, Image processing, Object-based image analysis, Pinyon-juniper

## Abstract

Pinyon and juniper expansion into sagebrush ecosystems is one of the major challenges facing land managers in the Great Basin. Effective pinyon and juniper treatment requires maps that accurately and precisely depict tree location and degree of woodland development so managers can target restoration efforts for early stages of pinyon and juniper expansion. However, available remotely sensed layers that cover a regional spatial extent lack the spatial resolution or accuracy to meet this need. Accuracy can be improved using object-based image analysis methods such as automated feature extraction, which has proven successful in accurately classifying land cover at the site-level but to date has yet to be applied to regional extents due to time and computational limitations. Using Feature Analyst™, we implement our framework with 1-m^2^ reference imagery provided by National Agricultural Imagery Program to classify conifers across Nevada and northeastern California. Our resulting binary conifer map has an overall accuracy of 86%. We discuss the advantages to accuracy and precision our framework provides compared to other classification methods.

● This framework allows automated feature extraction for large quantities of data and very high spatial resolution imagery

● It leverages supervised learning

● It results in high accuracy maps for regional spatial extents

## Graphical abstract

**Figure:**
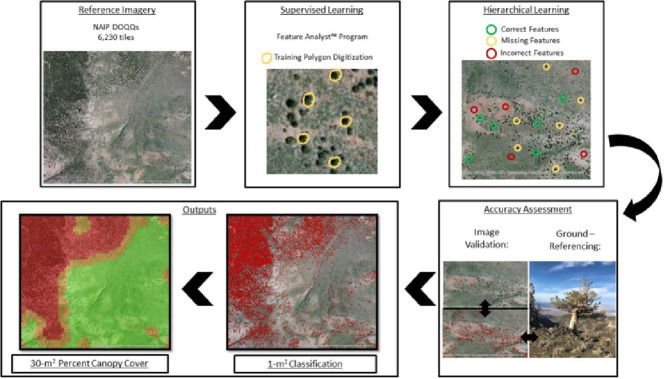

Specifications Table

**Table:** 

Subject Area	Agricultural and Biological Sciences
More specific subject area	Ecosystem management
Method name	A framework for automated regional conifer extraction
Name and reference of original method	Feature Analyst: Overwatch. Feature Analyst Version 5.1.2.0 for ArcGIS: Overwatch Systems Ltd., 2013.
Resource availability	ESRI, ArcGIS Desktop: Release 10.2: Environmental Systems Research Institute, 2013,
	Google. Google Earth Version 7.1.2.2041: Google Inc., 2013.
	Leica Geosystems, ERDAS Imagine: Version 13.00.0002: Leica Geosystems Geospatial Imaging LLC., 2013
	Overwatch. Feature Analyst Version 5.1.2.0 for ArcGIS: Overwatch Systems Ltd., 2013.
	USDA (U.S. Department of Agriculture). National Agriculture Imagery Program (NAIP) digital orthophoto quarter quads, 2013. https://gdg.sc.egov.usda.gov/ (Accessed November 2013)

## Regional mapping applications

**Fig. 1:**
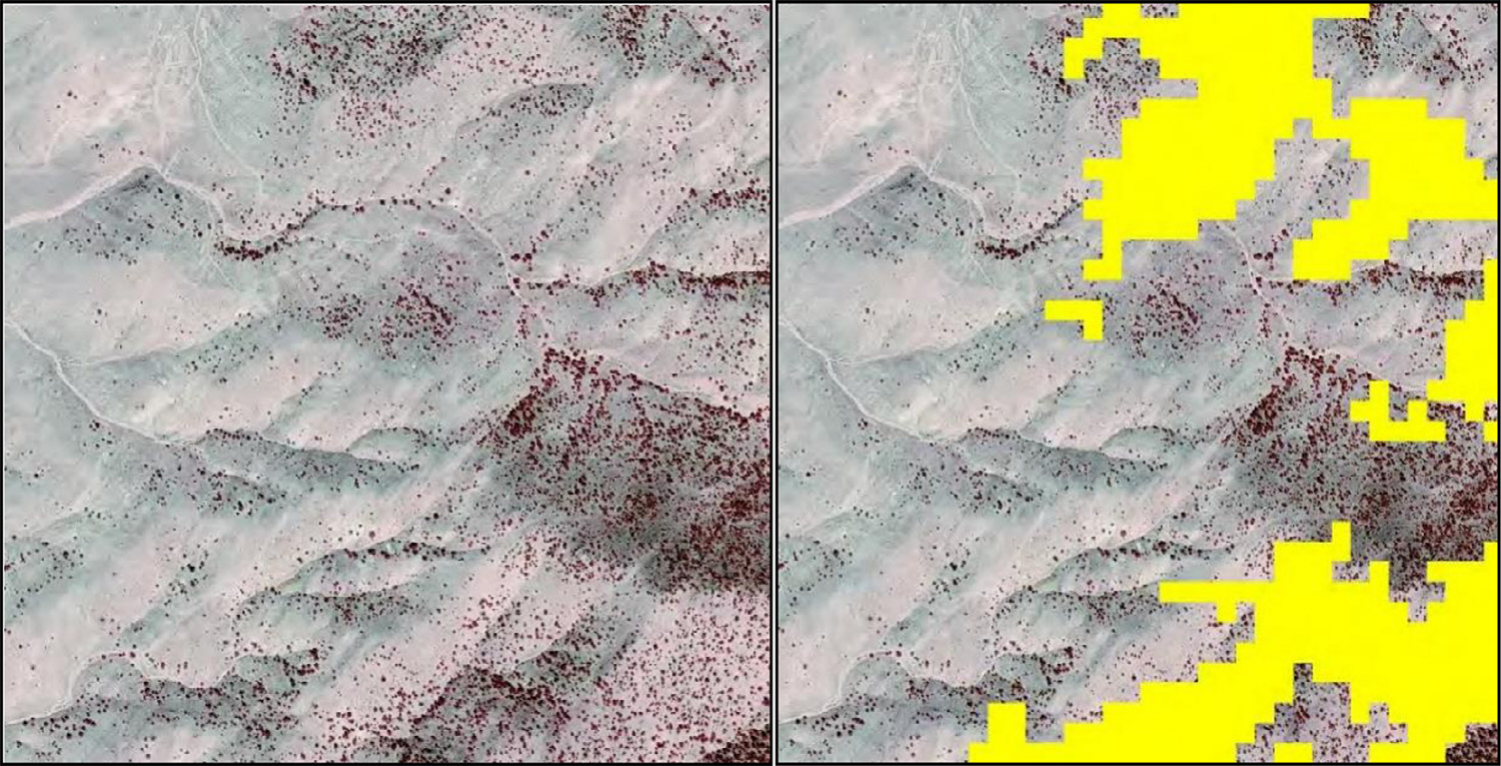
Pinyon-juniper in false-color, 1-m^2^ resolution National Agricultural Imagery Program (left) and Landsat- derived 900-m^2^ resolution pinyon-juniper class overlay (in yellow, right).

Improved management of sagebrush ecosystems in the Great Basin requires high resolution, high accuracy maps of pinyon and juniper (hereafter, conifer) distribution and density (measured as percent canopy cover) to identify potential treatment areas [8]. However, contemporary remote sensing products lack the resolution to provide accurate conifer locations and to identify early-phase woodland expansion (Fig. 1), which are crucial in planning targeted conifer treatment [8]. We applied object-based image analysis (OBIA) to map conifers at a resolution of 1-m^2^ using National Agriculture Imagery Program (NAIP; [15]) imagery collected in 2010 and 2013 as our reference data and the Feature Analyst™ toolbox [13] for Esri® ArcGIS™ Desktop ([5], Release 10.2, Redlands, California). Feature Analyst™ is an automated feature extraction (AFE) method that semi-automates the extraction of target features using a machine learning algorithm trained to delineate image objects based on the spectral and spatial signatures of defined cell neighborhoods [12]. AFE outperforms pixel-based methods [17] and is recognized as one of the most accurate OBIA methods available [12]. However, the user investment and computational requirements of AFE has restricted its use in regional mapping applications. We present a new framework for conducting broad-scale feature extraction using very high spatial resolution (VHSR) imagery that preserves the benefits of high user involvement algorithms, like improved classification, while optimizing efficiency with batch geospatial processing. We extracted conifer (mostly pinyon-juniper; however, we could not differentiate among species) image objects to create 1-m^2^ resolution binary conifer rasters across our study extent. We assessed mapping accuracy by analyzing errors of omission and commission using reference imagery and calculating overall accuracy for our mapping product. Finally, we qualitatively discuss how our AFE-based results compare with those derived recently using other techniques for high resolution mapping of conifers in rangeland ecosystems [6]. The resulting classification products are presented by Gustafson et al. [8] with accompanying descriptions of their significance to management applications.

## Study area

**Fig. 2:**
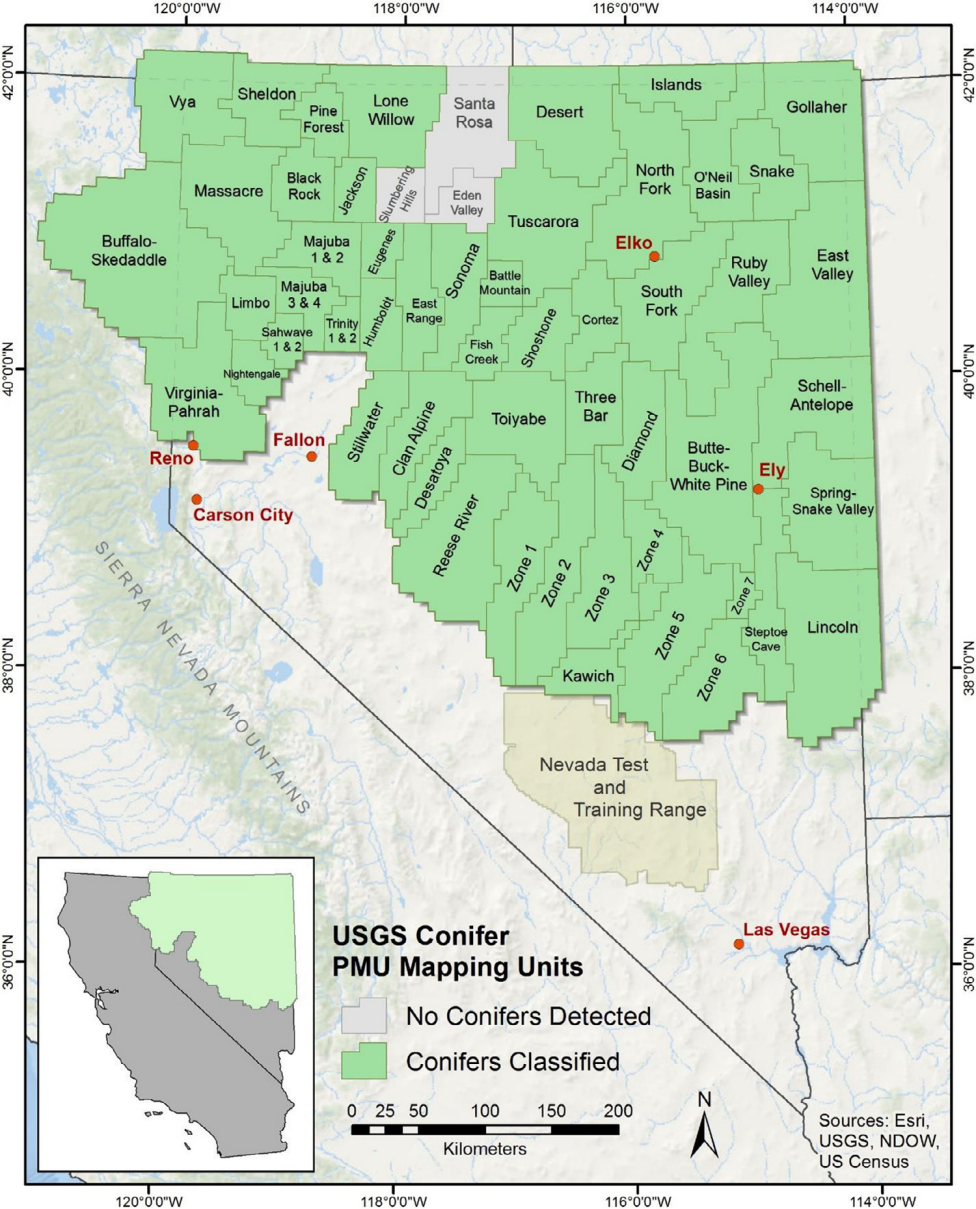
Mapping extent modified sage-grouse Population Management Units across Nevada and northeastern California.

Conifer mapping was conducted for all 61 Nevada Department of Wildlife sage-grouse Population Management Units (PMU; Fig. 2), as pinyon-juniper treatment is significantly motivated by sage-grouse habitat restoration [3]. We used the NAIP digital orthophoto quarter quads (DOQQs) because they are freely available, orthorectified, VHSR (<4- m^2^) products that comprise four spectral bands (three visible-light bands [RGB] and a near-infrared band), which allow classification of vegetation types, including conifers. Our study extent included 6,230 DOQQs from Nevada, and California along with small areas along the state borders of Oregon, Idaho and Utah that fell within the buffered PMU boundary. We buffered the extent of the PMUs by 10 kilometers (km) to prevent inaccurate moving window (or neighborhood) calculations within the study area along boundaries where "No Data" values would occur. We selected this size of buffer because sage-grouse typically do not use habitat more than 8 km from lek locations [2], [9]. A small section of our study area along the southern boundary was truncated by the Nevada Military Test and Training Range where the NAIP imagery was unavailable or redacted. We analyzed PMUs on a tile-by-tile basis by intersecting polygon boundaries of the DOQQs with the PMUs because inconsistencies among DOQQs (e.g., varying image quality, changes in lighting, inconsistent spectral values, shadows, parallax, and processing artifacts) required independent analysis of each tile for greatest classification accuracy. We further divided larger PMUs into smaller, more manageable zones. Zone boundaries followed DOQQ boundary polygons and were selected in low to non-conifer areas to minimize the potential for seamlines in classifications.

## Automated feature extraction

We individually reviewed each tile for the presence of conifers and processed tiles with conifers using the Feature Analyst™ Supervised Learning Wizard, which applies a supervised learning algorithm that extracts features meeting spectral and contextual specifications via a set of training polygons. We digitized a representative sample of conifer image objects across the entire tile to create this polygon training set. We distinguished conifers from other vegetation based on the hue of red under traditional false-color settings, and verified identification using Google Earth™ (version 7.1.2.2041 2013)[7]. We digitized clearly identifiable individual conifers for the training polygons to ensure the classification of isolated trees in low canopy density areas and used the spectral properties of the cells under false-color settings to delineate the conifer crown. This process trained the OBIA algorithm to distinguish trees from shadows and other vegetation types, minimizing misclassification. Each digitized image object consisted of at least three cells, a canopy area that corresponds to tree height (≥ 3m) readily tall enough to break through the sagebrush canopy. The number of samples per tile varied according to image quality and color variation, but always consisted of a minimum of five training polygons. We then used the training set as the input for Feature Analyst™’s Supervised Learning Wizard to generate conifer features from the four-band NAIP image.

In Feature Analyst™’s Supervised Learning Wizard, we specified parameters that best represented trees in the supervised machine learning algorithm. We defined our search neighborhoods using the "Natural Feature" feature selector with the "Bullseye 3" parameter over a 25-m^2^ moving window to define the spatial and spectral context used by the algorithm to extract features. "Natural Shape" discouraged the algorithm from using hard lines to generate image objects. The Bullseye search window reduced processing time by reducing the number of cells supplied to the learning algorithm while still representing the neighborhood. We specified a "pattern width" of 5 m to match the Bullseye moving window, and we chose 5 m in both instances because this width was larger than our minimum mapping unit, allowing the algorithm to discern features based on collective spectral qualities while still small enough to extract individual trees. Additionally, it allowed us to exclude smaller vegetation with similar spectral signatures and reduce the chance for errors of commission. Detected features > 3-m^2^ were aggregated. These parameters were determined to best classify trees after several rounds of tests.

We checked output conifer features for accuracy against the NAIP reference imagery. Typically, the initial results would be over- or under-classified. If the results were under-classified, more training polygons were added to the initial set and a new supervised classification was performed to replace the initial output. In some cases where a single learning algorithm could not be trained to recognize all conifer features (i.e., extremely dense stands), multiple algorithms were run on the same tile to target the variety of feature types and these outputs were merged together. If the results were over-classified, we digitized samples of incorrect, correct, and missed features, which were incorporated into a hierarchical supervised classification performed on the previous OBIA output. This hierarchical classification continued until misclassification errors were minimized based on visual comparison of feature outputs to the NAIP reference imagery. The "Hierarchical Learning" process in Feature Analyst™ is comprised of three sub-processes that retrain the algorithm and optimize the original output: (1) incorrect features are digitized for removal using the "Begin Removing Clutter Tool." We included removal polygon samples of all possible misclassifications such as shadows, riparian vegetation, and other non-conifer features. (2) The correct output features are then identified to retrain the OBIA algorithm. (3) Features that were missed by the previous supervised learning run are digitized and added to the training set using the "Begin Adding Missed Features Tool." After the hierarchical learning process was complete, we carried out additional geoprocessing to dissolve overlapping features, repair polygon geometry, and remove features < 3-m^2^. Any large misclassifications that occurred as a result of spectral overlap (e.g., algae in standing water, irrigated agricultural fields, wet meadows, riparian areas, or patches of other non-conifer vegetation) were removed using custom polygon masks. We then converted the clean shapefiles for each tile to 2- bit, binary VHSR rasters (1-m^2^).

We carried out several post-processing steps on output feature layers for each tile to further improve results. We reviewed tiles for obvious errors of omission and commission. We also checked against neighboring tiles for acute seamlines, which were signs of classification disagreement resulting from tile-based analysis. Tiles were iteratively re-analyzed if seamlines were pervasive. Raster output was considered ‘clean’ at the completion of this process, and then mosaicked for each zone or PMU in ERDAS Imagine (2013, Leica Geosystems, Atlanta, Georgia) using the "Automatic Most Nadir Seam" setting to minimize seamlines by overlapping areas where the distance to the center point of each image is equal.

## Accuracy assessment

**Fig. 3:**
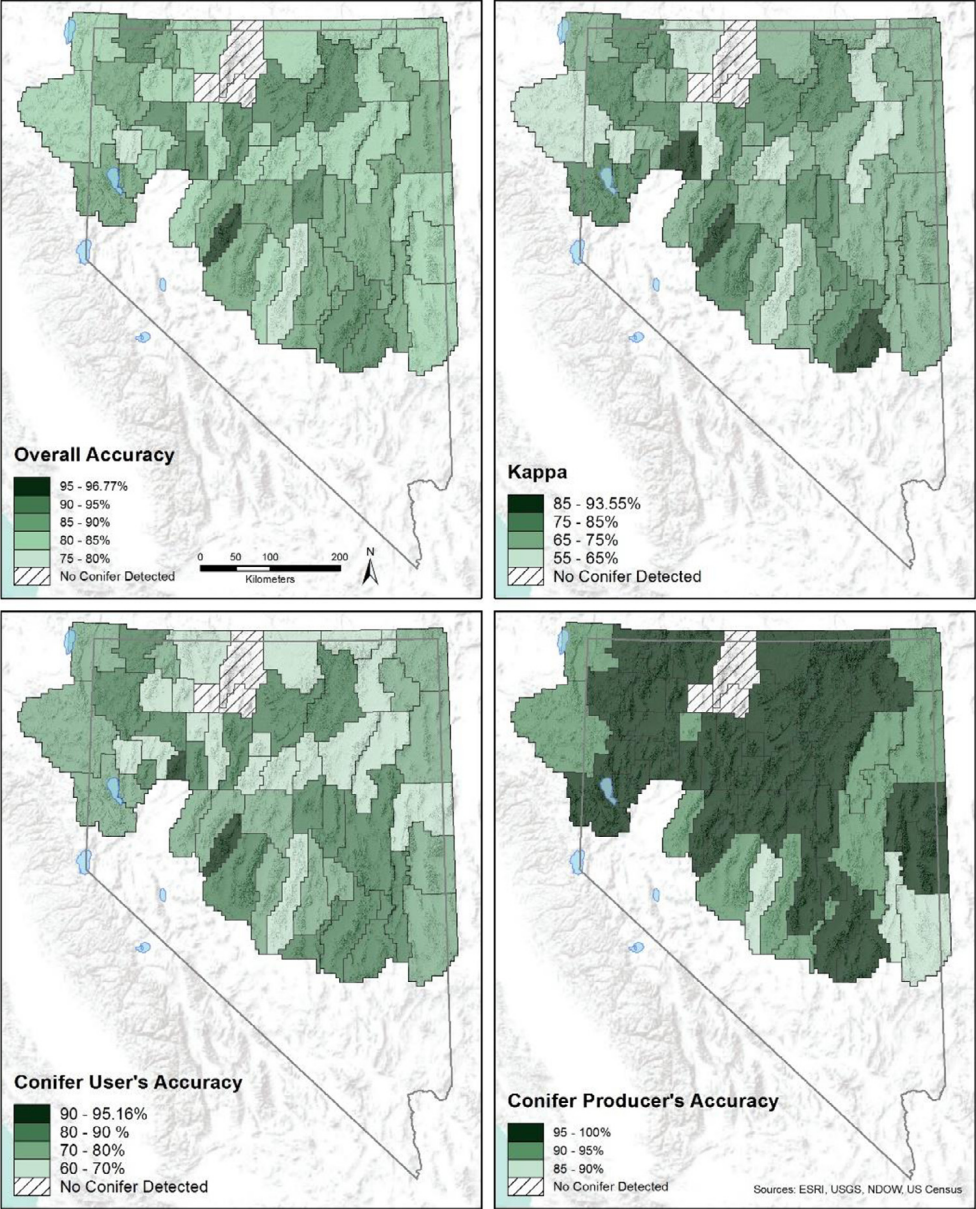
Maps showing overall accuracy (top left), Kappa statistic (top right), user's accuracy (error of commission; bottom left), and producer's accuracy (error of omission; bottom right) results by population management unit of mapped conifers at the 1-m^2^ resolution using automated feature

To assess the accuracy of OBIA conifer classification, we constructed an error matrix [4] for each PMU. We first generated stratified random points within the 1-m^2^ conifer and non-conifer classes and compared our classification at those locations against NAIP reference imagery. We standardized the number of points by sampling 100 points per the average classified area (km^2^) of the PMUs. We then divided the area of each PMU (km^2^) by this km^2^-per-point value to weight the number of random points generated for each PMU by its area, with a required minimum of 25 points generated for each PMU. Each random point was visually inspected for errors of omission (e.g., failing to identify a conifer) and commission (e.g., incorrectly classifying non-conifer as conifer) and the results entered in the error matrix (Table 1). We calculated the overall accuracy of conifer and non- conifer classification in each PMU, which identifies the percent of correct classifications from the total cases examined (Table 2; Fig. 3). To investigate bias in the OBIA towards errors of commission or omission, we also calculated the user's and producer's accuracy, respectively (Table 2,Fig. 3). The user's accuracy evaluates the reliability of the output conifer class by determining the percentage of cases correctly attributed to each class and the performance of the classification algorithm by identifying the percent detection of all cases in each class, respectively. The values in the confusion matrices were used to perform an estimated accuracy coefficient (kappa) analysis (Table 2; [4]). The kappa analysis generates the kappa coefficient ($K_{hat}$), which represents the percent accuracy adjusted for correct classification due to random chance. A $K_{hat}$> 60% indicate substantial agreement between classification and truth, and those > 80% are almost perfect [10].

**Table 1:** Error matrices by PMU used to calculate user's, producer's, and overall accuracy conifers mapped at a 1-m^2^ resolution across all using intensive Automated Feature Extraction (AFE) methods [*Khat*, estimated accuracy coefficient]

**Battle Mountain**					
	Conifer	Non-conifer	Total	User's accuracy	
Conifer random	17	8	25	68.00%	
Non-conifer random	0	25	25	100.00%	
Total	17	33	50		
Producer's accuracy	100.00%	75.76%		84.00%	Overall accuracy
				68.00%	K_hat_

**Black Rock**					
	Conifer	Non-conifer	Total	User's accuracy	
Conifer random	17	8	25	68.00%	
Non-conifer random	0	25	25	100.00%	
Total	17	33	50		
Producer's accuracy	100.00%	75.76%		84.00%	Overall accuracy
				68.00%	K_hat_

**Buffalo-Skedaddle**					
	Conifer	Non-conifer	Total	User's accuracy	
Conifer random	281	116	397	70.78%	
Non-conifer random	31	366	397	92.19%	
Total	312	482	794		
Producer's accuracy	90.06%	75.93%		81.49%	Overall accuracy
				62.97%	K_hat_

**Butte/Buck/White Pine**					
	Conifer	Non-conifer	Total	User's accuracy	
Conifer random	294	62	356	82.58%	
Non-conifer random	30	326	356	91.57%	
Total	324	388	712		
Producer's accuracy	90.74%	84.02%		87.08%	Overall accuracy
				74.16%	K_hat_

**Clan Alpine**					
	Conifer	Non-conifer	Total	User's accuracy	
Conifer random	91	25	116	78.45%	
Non-conifer random	1	115	116	99.14%	
Total	92	140	232		
Producer's accuracy	98.91%	82.14%		88.79%	Overall accuracy
				77.59%	K_hat_

**Cortez**					
	Conifer	Non-conifer	Total	User's accuracy	
Conifer random	42	18	60	70.00%	
Non-conifer random	1	59	60	98.33%	
Total	43	77	120		
Producer's accuracy	97.67%	76.62%		84.17%	Overall accuracy
				68.33%	K_hat_

**Desatoya**					
	Conifer	Non-conifer	Total	User's accuracy	
Conifer random	59	3	62	95.16%	
Non-conifer random	1	61	62	98.39%	
Total	60	64	124		
Producer's accuracy	98.33%	95.31%		96.77%	Overall accuracy
				93.55%	K_hat_

**Desert**					
	Conifer	Non-conifer	Total	User's accuracy	
Conifer random	17	8	25	68.00%	
Non-conifer random	0	25	25	100.00%	
Total	17	33	50		
Producer's accuracy	100.00%	75.76%		84.00%	Overall accuracy
				68.00%	K_hat_

**Diamond**					
	Conifer	Non-conifer	Total	User's accuracy	
Conifer random	78	19	97	80.41%	
Non-conifer random	4	93	97	95.88%	
Total	82	112	194		
Producer's accuracy	95.12%	83.04%		88.14%	Overall accuracy
				76.29%	K_hat_

**East Range**					
	Conifer	Non-conifer	Total	User's accuracy	
Conifer random	44	25	69	63.77%	
Non-conifer random	2	67	69	97.10%	
Total	46	92	138		
Producer's accuracy	95.65%	72.83%		80.43%	Overall accuracy
				60.87%	K_hat_

**East Valley**					
	Conifer	Non-conifer	Total	User's accuracy	
Conifer random	171	45	216	79.17%	
Non-conifer random	14	202	216	93.52%	
Total	185	247	432		
Producer's accuracy	92.43%	81.78%		86.34%	Overall accuracy
				72.69%	K_hat_

**Eugenes**					
	Conifer	Non-conifer	Total	User's accuracy	
Conifer random	17	8	25	68.00%	
Non-conifer random	1	24	25	96.00%	
Total	18	32	50		
Producer's accuracy	94.44%	75.00%		82.00%	Overall accuracy
				64.00%	K_hat_

**Fish Creek**					
	Conifer	Non-conifer	Total	User's accuracy	
Conifer random	23	12	35	65.71%	
Non-conifer random	0	35	35	100.00%	
Total	23	47	70		
Producer's accuracy	100.00%	74.47%		82.86%	Overall accuracy
				65.71%	K_hat_

**Gollaher**					
	Conifer	Non-conifer	Total	User's accuracy	
Conifer random	138	49	187	73.80%	
Non-conifer random	10	177	187	94.65%	
Total	148	226	374		
Producer's accuracy	93.24%	78.32%		84.22%	Overall accuracy
				68.45%	K_hat_

**Humboldt**					
	Conifer	Non-conifer	Total	User's accuracy	
Conifer random	35	4	39	89.74%	
Non-conifer random	0	39	39	100.00%	
Total	35	43	78		
Producer's accuracy	100.00%	90.70%		94.87%	Overall accuracy
				89.74%	K_hat_

**Islands**					
	Conifer	Non-conifer	Total	User's accuracy	
Conifer random	55	24	79	69.62%	
Non-conifer random	0	79	79	100.00%	
Total	55	103	158		
Producer's accuracy	100.00%	76.70%		84.81%	Overall accuracy
				69.62%	K_hat_

**Jackson**					
	Conifer	Non-conifer	Total	User's accuracy	
Conifer random	21	9	30	70.00%	
Non-conifer random	1	29	30	96.67%	
Total	22	38	60		
Producer's accuracy	95.45%	76.32%		83.33%	Overall accuracy
				66.67%	K_hat_

**Kawich**					
	Conifer	Non-conifer	Total	User's accuracy	
Conifer random	47	11	58	81.03%	
Non-conifer random	4	54	58	93.10%	
Total	51	65	116		
Producer's accuracy	92.16%	83.08%		87.07%	Overall accuracy
				74.14%	K_hat_

**Limbo**					
	Conifer	Non-conifer	Total	User's accuracy	
Conifer random	21	14	35	60.00%	
Non-conifer random	0	35	35	100.00%	
Total	21	49	70		
Producer's accuracy	100.00%	71.43%		80.00%	Overall accuracy
				60.00%	K_hat_

**Lincoln**					
	Conifer	Non-conifer	Total	User's accuracy	
Conifer random	239	67	306	78.10%	
Non-conifer random	32	274	306	89.54%	
Total	271	341	612		
Producer's accuracy	88.19%	80.35%		83.82%	Overall accuracy
				67.65%	K_hat_

**Lone Willow**					
	Conifer	Non-conifer	Total	User's accuracy	
Conifer random	16	9	25	64.00%	
Non-conifer random	0	25	25	100.00%	
Total	16	34	50		
Producer's accuracy	100.00%	73.53%		82.00%	Overall accuracy
				64.00%	K_hat_

**Majuba 1-2**					
	Conifer	Non-conifer	Total	User's accuracy	
Conifer random	33	6	39	84.62%	
Non-conifer random	0	39	39	100.00%	
Total	33	45	78		
Producer's accuracy	100.00%	86.67%		92.31%	Overall accuracy
				84.62%	K_hat_

**Majuba 3-4**					
	Conifer	Non-conifer	Total	User's accuracy	
Conifer random	25	11	36	69.44%	
Non-conifer random	0	36	36	100.00%	
Total	25	47	72		
Producer's accuracy	100.00%	76.60%		84.72%	Overall accuracy
				69.44%	K_hat_

**Massacre**					
	Conifer	Non-conifer	Total	User's accuracy	
Conifer random	79	19	98	80.61%	
Non-conifer random	1	97	98	98.98%	
Total	80	116	196		
Producer's accuracy	98.75%	83.62%		89.80%	Overall accuracy
				79.59%	K_hat_

**Nightengale**					
	Conifer	Non-conifer	Total	User's accuracy	
Conifer random	19	6	25	76.00%	
Non-conifer random	0	25	25	100.00%	
Total	19	31	50		
Producer's accuracy	100.00%	80.65%		88.00%	Overall accuracy
				76.00%	K_hat_

**North Fork**					
	Conifer	Non-conifer	Total	User's accuracy	
Conifer random	61	13	74	82.43%	
Non-conifer random	0	74	74	100.00%	
Total	61	87	148		
Producer's accuracy	100.00%	85.06%		91.22%	Overall accuracy
				82.43%	K_hat_

**O'Neil**					
	Conifer	Non-conifer	Total	User's accuracy	
Conifer random	23	13	36	63.89%	
Non-conifer random	0	36	36	100.00%	
Total	23	49	72		
Producer's accuracy	100.00%	73.47%		81.94%	Overall accuracy
				63.89%	K_hat_

**Pine Forest**					
	Conifer	Non-conifer	Total	User's accuracy	
Conifer random	20	5	25	80.00%	
Non-conifer random	0	25	25	100.00%	
Total	20	30	50		
Producer's accuracy	100.00%	83.33%		90.00%	Overall accuracy
				80.00%	K_hat_

**Reese River**					
	Conifer	Non-conifer	Total	User's accuracy	
Conifer random	178	39	217	82.03%	
Non-conifer random	13	204	217	94.01%	
Total	191	243	434		
Producer's accuracy	93.19%	83.95%		88.02%	Overall accuracy
				76.04%	K_hat_

**Ruby Valley**					
	Conifer	Non-conifer	Total	User's accuracy	
Conifer random	120	60	180	66.67%	
Non-conifer random	7	173	180	96.11%	
Total	127	233	360		
Producer's accuracy	94.49%	74.25%		81.39%	Overall accuracy
				62.78%	K_hat_

**Sahwave 1-2**					
	Conifer	Non-conifer	Total	User's accuracy	
Conifer random	22	3	25	88.00%	
Non-conifer random	1	24	25	96.00%	
Total	23	27	50		
Producer's accuracy	95.65%	88.89%		92.00%	Overall accuracy
				84.00%	K_hat_

**Schell-Antelope**					
	Conifer	Non-conifer	Total	User's accuracy	
Conifer random	149	64	213	69.95%	
Non-conifer random	5	208	213	97.65%	
Total	154	272	426		
Producer's accuracy	96.75%	76.47%		83.80%	Overall accuracy
				67.61%	K_hat_

**Sheldon**					
	Conifer	Non-conifer	Total	User's accuracy	
Conifer random	49	9	58	84.48%	
Non-conifer random	0	58	58	100.00%	
Total	49	67	116		
Producer's accuracy	100.00%	86.57%		92.24%	Overall accuracy
				84.48%	K_hat_

**Shoshone**					
	Conifer	Non-conifer	Total	User's accuracy	
Conifer random	40	20	60	66.67%	
Non-conifer random	1	59	60	98.33%	
Total	41	79	120		
Producer's accuracy	97.56%	74.68%		82.50%	Overall accuracy
				65.00%	K_hat_

**Snake**					
	Conifer	Non-conifer	Total	User's accuracy	
Conifer random	53	17	70	75.71%	
Non-conifer random	2	68	70	97.14%	
Total	55	85	140		
Producer's accuracy	96.36%	80.00%		86.43%	Overall accuracy
				72.86%	K_hat_

**Sonoma**					
	Conifer	Non-conifer	Total	User's accuracy	
Conifer random	46	9	55	83.64%	
Non-conifer random	0	55	55	100.00%	
Total	46	64	110		
Producer's accuracy	100.00%	85.94%		91.82%	Overall accuracy
				83.64%	K_hat_

**South Fork**					
	Conifer	Non-conifer	Total	User's accuracy	
Conifer random	114	55	169	67.46%	
Non-conifer random	3	166	169	98.22%	
Total	117	221	338		
Producer's accuracy	97.44%	75.11%		82.84%	Overall accuracy
				65.68%	K_hat_

**Spring-Snake Valley**					
	Conifer	Non-conifer	Total	User's accuracy	
Conifer random	140	52	192	72.92%	
Non-conifer random	7	185	192	96.35%	
Total	147	237	384		
Producer's accuracy	95.24%	78.06%		84.64%	Overall accuracy
				69.27%	K_hat_

**Steptoe-Cave**					
	Conifer	Non-conifer	Total	User's accuracy	
Conifer random	93	18	111	83.78%	
Non-conifer random	11	100	111	90.09%	
Total	104	118	222		
Producer's accuracy	89.42%	84.75%		86.94%	Overall accuracy
				73.87%	K_hat_

**Stillwater**					
	Conifer	Non-conifer	Total	User's accuracy	
Conifer random	37	12	49	75.51%	
Non-conifer random	4	45	49	91.84%	
Total	41	57	98		
Producer's accuracy	90.24%	78.95%		83.67%	Overall accuracy
				67.35%	K_hat_

**Three Bar**					
	Conifer	Non-conifer	Total	User's accuracy	
Conifer random	84	13	97	86.60%	
Non-conifer random	4	93	97	95.88%	
Total	88	106	194		
Producer's accuracy	95.45%	87.74%		91.24%	Overall accuracy
				82.47%	K_hat_

**Toiyabe**					
	Conifer	Non-conifer	Total	User's accuracy	
Conifer random	109	41	150	72.67%	
Non-conifer random	2	148	150	98.67%	
Total	111	189	300		
Producer's accuracy	98.20%	78.31%		85.67%	Overall accuracy
				71.33%	K_hat_

**Trinity 1-2**					
	Conifer	Non-conifer	Total	User's accuracy	
Conifer random	23	2	25	92.00%	
Non-conifer random	1	24	25	96.00%	
Total	24	26	50		
Producer's accuracy	95.83%	92.31%		94.00%	Overall accuracy
				88.00%	K_hat_

**Tuscarora**					
	Conifer	Non-conifer	Total	User's accuracy	
Conifer random	21	4	25	84.00%	
Non-conifer random	0	25	25	100.00%	
Total	21	29	50		
Producer's accuracy	100.00%	86.21%		92.00%	Overall accuracy
				84.00%	K_hat_

**Virginia-Pahrah**					
	Conifer	Non-conifer	Total	User's accuracy	
Conifer random	139	39	178	78.09%	
Non-conifer random	4	174	178	97.75%	
Total	143	213	356		
Producer's accuracy	97.20%	81.69%		87.92%	Overall accuracy
				75.84%	K_hat_

**Vya**					
	Conifer	Non-conifer	Total	User's accuracy	
Conifer random	128	49	177	72.32%	
Non-conifer random	12	165	177	93.22%	
Total	140	214	354		
Producer's accuracy	91.43%	77.10%		82.77%	Overall accuracy
				65.54%	K_hat_

**Zone 1 (Monitor)**					
	Conifer	Non-conifer	Total	User's accuracy	
Conifer random	80	26	106	75.47%	
Non-conifer random	10	96	106	90.57%	
Total	90	122	212		
Producer's accuracy	88.89%	78.69%		83.02%	Overall accuracy
				66.04%	K_hat_

**Zone 2 (Monitor)**					
	Conifer	Non-conifer	Total	User's accuracy	
Conifer random	101	62	163	61.96%	
Non-conifer random	6	157	163	96.32%	
Total	107	219	326		
Producer's accuracy	94.39%	71.69%		79.14%	Overall accuracy
				58.28%	K_hat_

**Zone 3 (Monitor)**					
	Conifer	Non-conifer	Total	User's accuracy	
Conifer random	110	39	149	73.83%	
Non-conifer random	3	146	149	97.99%	
Total	113	185	298		
Producer's accuracy	97.35%	78.92%		85.91%	Overall accuracy
				71.81%	K_hat_

**Zone 4 (Monitor/Quinn)**					
	Conifer	Non-conifer	Total	User's accuracy	
Conifer random	40	13	53	75.47%	
Non-conifer random	0	53	53	100.00%	
Total	40	66	106		
Producer's accuracy	100.00%	80.30%		87.74%	Overall accuracy
				75.47%	K_hat_

**Zone 5 (Quinn)**					
	Conifer	Non-conifer	Total	User's accuracy	
Conifer random	130	23	153	84.97%	
Non-conifer random	1	152	153	99.35%	
Total	131	175	306		
Producer's accuracy	99.24%	86.86%		92.16%	Overall accuracy
				84.31%	K_hat_

**Zone 6 (Quinn)**					
	Conifer	Non-conifer	Total	User's accuracy	
Conifer random	124	17	141	87.94%	
Non-conifer random	3	138	141	97.87%	
Total	127	155	282		
Producer's accuracy	97.64%	89.03%		92.91%	Overall accuracy
				85.82%	K_hat_

**Zone 7 (Quinn)**					
	Conifer	Non-conifer	Total	User's accuracy	
Conifer random	22	3	25	88.00%	
Non-conifer random	2	23	25	92.00%	
Total	24	26	50		
Producer's accuracy	91.67%	88.46%		90.00%	Overall accuracy
				80.00%	K_hat_

**Table 2:** Accuracy results of mapping conifers at the 1-m^2^ resolution by population management unit using automated feature extraction methods within greater sage-grouse habitat of Nevada and California. [*Khat*, estimated accuracy coefficient. N/A, population management units where conifers were not detected].

Population management unit	Conifer user's accuracy (%)	Non-conifer user's accuracy (%)	Conifer producer's accuracy (%)	Non-conifer producer's accuracy (%)	Overall accuracy (%)	Khat (%)
Battle Mountain	68.00	100.00	100.00	75.76	84.00	68.00
Black Rock	68.00	100.00	100.00	75.76	84.00	68.00
Buffalo-Skedaddle	70.78	92.19	90.06	75.93	81.49	62.97
Butte/Buck/White Pine	82.58	91.57	90.74	84.02	87.08	74.16
Clan Alpine	78.45	99.14	98.91	82.14	88.79	77.59
Cortez	70.00	98.33	97.67	76.62	84.17	68.33
Desatoya	95.16	98.39	98.33	95.31	96.77	93.55
Desert	68.00	100.00	100.00	75.76	84.00	68.00
Diamond	80.41	95.88	95.12	83.04	88.14	76.29
East Valley	79.17	93.52	92.43	81.78	86.34	72.69
East Range	63.77	97.10	95.65	72.83	80.43	60.87
Eden Valley	N/A	N/A	N/A	N/A	N/A	N/A
Eugenes	68.00	96.00	94.44	75.00	82.00	64.00
Fish Creek	65.71	100.00	100.00	74.47	82.86	65.71
Gollaher	73.80	94.65	93.24	78.32	84.22	68.45
Humboldt	89.74	100.00	100.00	90.70	94.87	89.74
Islands	69.62	100.00	100.00	76.70	84.81	69.62
Jackson	70.00	96.67	95.45	76.32	83.33	66.67
Kawich	81.03	93.10	92.16	83.08	87.07	74.14
Limbo	60.00	100.00	100.00	71.43	80.00	60.00
Lincoln	78.10	89.54	88.19	80.35	83.82	67.65
Lone Willow	64.00	100.00	100.00	73.53	82.00	64.00
Majuba 1 and 2	84.62	100.00	100.00	86.67	92.31	84.62
Majuba 3 and 4	69.44	100.00	100.00	76.60	84.72	69.44
Massacre	80.61	98.98	98.75	83.62	89.80	79.59
Nightengale	76.00	100.00	100.00	80.65	88.00	76.00
North Fork	82.43	100.00	100.00	85.06	91.22	82.43
O'Neil Basin	63.89	100.00	100.00	73.47	81.94	63.89
Pine Forest	80.00	100.00	100.00	83.33	90.00	80.00
Reese River	82.03	94.01	93.19	83.95	88.02	76.04
Ruby Valley	66.67	96.11	94.49	74.25	81.39	62.78
Sahwave 1 and 2	88.00	96.00	95.65	88.89	83.80	84.00
Santa Rosa	N/A	N/A	N/A	N/A	N/A	N/A
Schell/Antelope	69.95	97.95	96.75	76.47	83.80	67.61
Sheldon	84.48	100.00	100.00	86.57	92.24	84.48
Shoshone	66.67	98.33	97.56	74.68	82.50	65.00
Slumbering Hills	N/A	N/A	N/A	N/A	N/A	N/A
Snake	75.71	97.14	96.36	80.00	86.43	72.86
Sonoma	83.64	100.00	100.00	85.94	91.82	83.64
South Fork	67.46	98.22	97.44	75.11	82.84	65.68
Steptoe Cave	83.78	90.09	89.42	84.75	86.94	73.87
Stillwater	75.51	91.84	90.24	78.95	83.67	67.35
Three Bar	86.60	95.88	95.45	87.74	91.24	82.47
Toiyabe	72.67	98.67	98.20	78.31	85.67	71.33
Trinity 1 and 2	92.00	96.00	95.83	92.31	94.00	88.00
Tuscarora	84.00	100.00	100.00	86.21	92.00	84.00
Virginia-Pahrah	78.09	97.75	97.20	81.69	87.92	75.84
Vya	72.32	93.22	91.43	77.10	82.77	65.54
Zone 1 (Monitor)	75.47	90.57	88.89	78.69	83.02	66.04
Zone 2 (Monitor)	61.96	96.32	94.39	71.69	79.14	58.28
Zone 3 (Monitor)	73.83	97.99	97.35	78.92	85.91	71.81
Zone 4 (Monitor and Quinn)	75.47	100.00	100.00	80.30	87.74	75.47
Zone 5 (Quinn)	84.97	99.35	99.24	86.86	92.16	84.31
Zone 6 (Quinn)	87.94	97.87	97.64	89.03	92.91	85.82
Zone 7 (Quinn)	88.00	92.00	91.67	88.46	90.00	80.00
Spring-Snake Valley	72.92	96.35	95.24	78.06	84.64	69.27

## Framework benefits

Our analysis framework utilized intensive AFE to classify target features across an entire region from VHSR imagery that resulted in comprehensive and highly accurate outputs (Table 2). OBIA methods like AFE that require higher levels of automation are known to be time consuming [1], [6], [14], which has restricted the scope of their application [1], [11]. Our framework reduces processing time and computational demand in order to make the implementation of such OBIA methods feasible across relatively large spatial extents. This reduction was primarily accomplished by leveraging user-friendly, semi-automated, and inductive learning algorithms in Feature Analyst™to decrease user investment and processing time [11], [12]. We also took advantage of parameters such as the bullseye pattern to reduce the amount of data processed [12]. However, AFE and other semi- automated OBIA methods require substantial operator investment ([14], [16]; Falkowski and Evans, 2012). For example, to produce our conifer classification, each tile required individualized training polygon development and supervised (often hierarchical) learning runs. The volume of work necessary to map the state with our framework required 10 analysts working congruently for several months.

The high operator-involvement of Feature Analyst™ promotes more accurate classification [12] but decreases reproducibility and increases analysis time [14]. To balance these tradeoffs, we integrated our Feature Analyst™ workflow with many time- saving geoprocessing steps such as analyzing imagery on a tile-by-tile basis to reduce computation time and allow for simultaneous, customized AFE, and performing validation within PMUs. Geoprocessing steps such as rasterization and mosaicking were iterated within PMUs in ArcGIS using Model Builder ([5], Release 10.2, Redlands, California) so that multiple classified tiles could undergo post-processing in quick succession and simultaneously. We also used Model Builder to iterate the calculation and reclassification of percent canopy cover smoothed by the 50-m radius neighborhood within sectors. Our framework also allows for several time saving mechanisms going forward. For example, post-processing could be further automated by iterating across PMUs and sectors. User-investment could be reduced on the front end by mosaicking the NAIP tiles into a single layer. However, analysis of VHSR imagery at such a large spatial extent remains limited by processing power. Also, Feature Analyst™ has several features that facilitate further automation via batch processing, such as the ability to save training polygons and learning algorithms for repeated use, allowing analysts to use a single training set and model for all imagery and greatly reduce the user-investment and processing time required for each tile [12]. We could not use these functions largely because of inconsistent quality of NAIP tiles across our mapping extent. However, such features are available for future applications and could easily be incorporated into this existing framework.

## Declaration of Competing Interest

The authors declare that they have no known competing financial interests or personal relationships that could have appeared to influence the work reported in this paper.

## Appendix Supplementary materials

Image, application 1
