# Supplementary material for: A customized framework for regional classification of conifers using automated feature extraction
Source: MethodsX. 2021 May 10;8:101379. doi: 10.1016/j.mex.2021.101379 (PMC8374470; doi:10.1016/j.mex.2021.101379)
Supplement: Supplementary file 1 [file mmc1.docx]

**Supplementary material *and/or* Additional information:**

**Spatial Wavelet Analysis Comparison**

We chose to use AFE as our method of OBIA because of its reported accuracy (O’Brien, 2003; Bruce, 2008; Opitz and Blundell, 2008; Tsai et al., 2011), yet Spatial Wavelet Analysis (SWA, another type of OBIA) has been successfully employed for a number of conifer classification products (Falkowski et al., 2006; Strand et al., 2006, 2008; Falkowski and Evans, 2012). Most recently, Falkowski et al. (2017) used SWA to produce high-resolution (1-m^2^) canopy cover classification of conifers across an 11-State region that included the Great Basin. SWA extracts conifers from imagery by convolving various sized two-dimensional Mexican Hat Wavelet functions, which have a circular shape that emulates tree crowns, to capture variability in tree crown dimension (Table 3). The output is a circular feature at the location of the detected tree with a diameter specified by the size of the best-fitting wavelet. Like AFE, there are tradeoffs among object extraction speed, mapping extent, and accuracy using SWA to classify canopy cover under different conditions (Table 3). Because SWA classifies image objects by signal size and shape, it can confuse background features such as roads or streambeds for target features, and it cannot readily differentiate among vegetation types, so it is biased towards errors of commission that require manual correction (Strand et al., 2006; Falkowski and Evans, 2012; Falkowski et al. 2017). Although formal testing is required, AFE may not share this bias because it uses similarities in the focal pixel cell and training image object spectral signatures to segment target features rather than signal and wavelet pattern. Also, SWA is prone to under-classifying dense canopy areas (Strand et al., 2006; Falkowski et al., 2017) because the wavelet loses the ability to recognize trees as a target feature when the shape of the signal is no longer circular (Strand et al., 2008). Accordingly, SWA may not be appropriate in closed canopy systems where canopy cover exceeds 40% (Falkowski et al., 2017). In contrast, our application of AFE in Feature Analyst™ does not have a high rate of omission errors in the higher conifer canopy classes (3%) largely because the algorithm can be parameterized to segment image objects by targeted spectral properties rather than shape. However, AFE will return dense canopies as a single object that precludes identification of individual trees.

One of the greatest advantages of using SWA is its multiscale functionality (Strand et al., 2006; Falkowski et al., 2017; Table 3). However, we used known characteristics of mature conifer canopy area to determine the correct scale of the analysis. We ultimately selected the threshold scale of 25-m^2^ because it covered our 3-m^2^ cut-off for minimum conifer area, and it returned the highest accuracy output in our initial tests. Also, the 25-m^2^ restriction allowed us to exclude many non-target species that are spectrally inseparable from conifers. This criterion inherently omitted some small conifers, but these trees are estimated to represent a very small percentage of woody biomass (Strand et al., 2008), and we opted to reduce errors of commission to provide a more conservative and overall accurate classification. In contrast, SWA may misclassify other species as conifers because they meet requisite size and shape criteria. Therefore, SWA may overestimate conifer cover in low to no cover areas such as riparian zones and salt flats dominated by other woody plant species (Table 3). Accurate classification of conifers in low canopy cover areas is critical because sage-grouse are sensitive to small percentage increases in canopy cover, and overestimation can mask regions that represent ecological traps for sage-grouse and could be targeted for pinyon-juniper removal (Baruch-Mordo et al., 2013; Coates et al., 2017).

**Table 3.**  Comparison of Spatial Wavelet Analysis and Automated Feature Extraction for identifying conifers.

____________________________________________________________________________________

|  | **Spatial Wavelet Analysis** | **Automated Feature Extraction** |
| --- | --- | --- |
|  |  |  |
| *Resolution* | 1-m^2^ | 1-m^2^ |
| *Computation Time* | Moderate | Slow |
| *Accuracy* | Unknown | >85% |
| *Method* | Mexican Hat Wavelet | Supervised and Hierarchical Learning |
| *Classification* | Size and Shape | Spectral Signature |
| *Scale* | Multiscale | Single, predefined scale |
| *Species ID* | No | No |
|  |  |  |

Nevertheless, AFE and SWA methods have been used to produce canopy cover products specifically to assist managers with sagebrush ecosystem and habitat restoration within the same region, and both methods have inherent advantages and disadvantages. Research that focuses more directly on comparing the two methods at large spatial extents would be highly beneficial, especially regarding: (1) assessment of differences in accuracy among canopy densities to define potential spatial discrepancies in performance; and (2) contrasting sources of commission errors to understand viability and future utility of these methods at different spatial extents. These comparisons may reveal that in some instances, SWA and AFE complement each other, whereas in other instances, one method out-performs the other.
